# Additional Clinical Cases

**Published:** 1893-02

**Authors:** B. Fincke

**Affiliations:** Brooklyn, N. Y.


### ADDITIONAL CLINICAL CASES.

B. Fincke, M. D., Brooklyn, N. Y.

Dr. Buchner, in München, retorted to an unbeliever in homœopathic potencies: "The ox does not believe either, and yet he is cured." As a counterpart to this historical homœopathic ox, the following cases show the unbelief of the homœopathic hen:

(1) A hen six years old, laying three to four eggs a week, began to lay eggs without the hard shell and ate them.

A dose of Calc. carb. 9c, would not remedy the evil in a week. But after another dose of Calc. carb. cm the hen laid a hard-shell egg the second day, and continued so ever since.

(2) When the hens get the chicken-cholera, about ten pellets of Veratrum alb. 9c, are dropped in their drink-water (three quarts), and in a few hours they are all right again.

(3) The hen sub 1 was at one time so constipated that we feared to lose her with her fruitless exertions. A dose of Nux-vom. 9c relieved her within an hour.

(4) A silver-spangled Hamburg got frightened and ran around in a narrow circle. A dose of Bellad. 9c restored her before an hour was passed, and she continued well, and was a capital layer for four years more.

(5) Hens with croup have frequently been saved by a dose of Spong. tosta. 9c, often in an incredibly short time.

The dose was given in this way: The hen was captured, and held by another person, the bill was opened with the left hand, and with the right hand a few pellets were poured from the bottle down the throat.

### Discussion.

Dr. Hastings—We have had for a number of years a very fine cat in our family. About five years ago it was reported that the cat was not eating anything at all. I found that somebody had clipped the smellers off close. The cat had refused to eat for three days, and had great signs of drooping. It occurred to me that these smellers were extremely sensitive, and probably supplied with nerves, and so I gave Hypericum, and in one hour she took her food.

Dr. Clark—I was asked to see a valuable English stallion that was troubled with roaring. He trotted about one hundred feet and came up with very loud breathing. I concluded to give him Sulphur, but after two weeks he was no better; but Brom.$^{500}$ cured him.

Dr. F. Powel—I have a patient who had a horse that had been over-driven. The urine was entirely suppressed, and the horse was in great agony. This condition is considered fatal. I decided that Hyosciamus was the remedy, and gave him four powders of the 200th. In six hours the urine came, and the horse recovered.

Dr. James—I have a mare that has always had very good health. I had made a stipulation at the stable that she was not to be treated or doctored with any medicine of any kind. About a year ago the horse did not come around to the office in the morning as usual, and soon after one of the stable hands came to say that the horse was very sick with colic. She had been taken about an hour before, and the stable hands had, contrary to orders, undertaken to treat her themselves with some quack stuff which they had. I found her lying on her back, groaning, in great agony, kicking her feet. When I spoke to her she scrambled to her feet, staggered toward me and fell down again. Her feet were all drawn together, all four feet together. This suggested the remedy Colocynth. I gave her some pellets. In five minutes she stopped groaning, in ten minutes she lay quiet, and in fifteen minutes she got up. The stableman said: "Great Scott! that must be a powerful opiate." He wanted some of that medicine right away.

Dr. Dever—When I was in Michigan, a man who had a fancy for dogs came to me and said that a dog of his had a swollen throat and was frothing at the mouth. A few doses of medicine cured the dog as readily as if he had been a human being, every bit as easy. No imagination there; neither was faith necessary to the cure.

Dr. Fincke—It is just forty years ago since I was on the ship coming to America. The captain told me there was a fine Newfoundland dog on board who was suffering from constipation. I gave a dose of Aconite$^{30}$, and it cured him.

Dr. Rushmore—I was told by a homœopathic veterinary surgeon that the best remedy for wind colic was Ammon-caust. in the 30th potency.

Dr. Taft—Last summer, before going to Richfield Springs, one of my patients asked me what he should do if his horse had the colic while I was away. I told him that while there was no specific for colic, I would do the best I could, and left him a bottle of Colocynth pellets. The horse did not have the colic at that time, but some time after I returned he did have it, and the Colocynth did not help. I was sent for and watched the animal for some time. I noticed that when a paroxysm of pain came the horse would rise and kick the stall viciously. I gave Nux-vom. and cured him very quickly. My own horse had the colic twice very severely. The stableman sent for me each time, and Colocynth did very rapid work. So much so that the stableman asked for a bottle of the medicine. I knew there must be something wrong with the horse to have colic twice in succession, and I remembered that for a few days before each attack, instead of walking up to the hitching-post, as usual, she would stand out in the middle of the road, and look and act for all the world like a stubborn child. I gave her Nux-vom., and she has never had a colic since.

Dr. Long—Has any one in the room ever cured a horse of blood or bone spavin?

Dr. Kimball—I cured one in my own mare. When the swelling appeared, the stableman blistered it without my knowledge, and the mare did not improve. I gave her Rhus, which helped, and afterward Phos-acid, which cured. The stableman was confident it was a bone spavin.

Dr. Farley—I have used Colchicum in overfed animals with disgust for food, and also Thuja, where the uncovered parts would sweat, both with success.

Dr. Taft—I had a case of a horse that was injured by a nail in the foot. The veterinary surgeon said it would die. I asked for the privilege of giving medicine, and gave it Ledum. A member of the family was taking lessons in Christian Science, and treated the horse at the same time. I do not know which of us deserves the credit, but the horse was cured.

Dr. Wesselhœft—I cured a mare of what is called poll evil. A veterinary told me that it was a disease which is never cured. I found a sore about three inches back of her ears discharging offensive pus. They told me I had better kill the horse. I didn't kill her but I did cure her. I put her into another stable with directions to give her a powder of Silicea$^{200}$, about once in two weeks. I saw the mare again in six weeks. The men around the stable said, "This mare is getting well." I stopped the medicine and she was entirely well in three months. She died last year at the ripe age of thirty-three. The abscess made no change in the position of the head and there was only a small depressed cicatrix on the left side of her neck. The pus was very offensive.

Dr. Kent—There can be no disputing these cures on the horse, and nothing proves so satisfactorily the genuine action of the potencies as these results on the dumb, unthinking animals. There is a trouble with the horse that is not very infrequent. The farriers call it blind staggers. It seems to be a form of vertigo. When the horse which I treated warmed up a little, he would tremble and stagger and seem perfectly blind. I administered a dose of Sulphur, which relieved him for a time only. Another dose was given him and it came back. The horse developed a quarter crack, which is a sign of defective nutrition. This led me to give a dose of Graphites, which cured both conditions. Not long ago I treated a horse for chills. The chill came on at irregular intervals, ranging from one to five in the afternoon. For these irregular chills I gave Arsenic$^{8m}$. I had to let the horse have several chills before I ascertained that Arsenic was the remedy. Of course we are compelled to prescribe on very meagre information in these cases. I have seen during the last year a tumor as big as my fist drop off from a single dose of Thuja$^{73m}$. It took about four weeks. Of course, as I am talking of horses, it was on a horse.

Dr. Kent—There is a symptom known as pointing in the horse. When the horse stops there seems to be some sort of soreness in the shoulder and the foot will be pointed outward. I have cured that condition a number of times with Ferrum when the left shoulder is the one affected.

Dr. Bell—Almost always horses coming from the country to Boston get a fever, with cough, profuse catarrh, and swelling of the glands in the neck. The remedy is Silicea; it cures them in almost every instance.

Dr. Jameson—I had a little experience in the animal line with a sick cow. She had just calved. She had high fever, eyes injected, respiration rapid, and abdomen distended. I gave her Bell.$^{200}$ every two hours. The owner came in the next morning and reported a change for the better about midnight, and in the morning she was quite well.

Dr. Baylies—Some months ago my horse was brought to the door with a loose shoe; in traveling tore it off, was lamed, and therefore confined to the stable five days. When I began to use him again I observed for a block a mincing gait of the hinder extremities; in a few minutes he was only able to walk and threatened to stop. I had great difficulty in getting him back to the stable, and sent for a veterinary. Before he arrived the horse was dragging himself about the stall in great agony, with the hind hoofs flexed; the region of the hips and sacrum was very much swollen, and this swelling and the paraplegia were increasing with wonderful rapidity. He would lie down and raise himself often only upon the forefeet, then drop again. Untrained to study the symptoms of horses except in the most ordinary ailments, I could not attempt treatment. I suspected acute disease of the lumbo-sacral portion of the spinal cord; the veterinary called it azoturia and attributed it to continuance of the usual quantity of food, especially of oats, while confined to the stall; fever was high, the urine turbid and of dark brownish color, and strong ammoniacal odor. He gave him Aconite tincture every four hours, and an enema containing Aloes. The horse steadily improved and recovered after about four weeks in the stable (in a box stall) and nearly two months in pasture, and is himself again. The pathology of this disease appears not fully understood.

Dr. E. E. Case—I was unfortunate enough to have five cases of scarlatina in my house. To destroy the bedding, I put it in a wagon, took it away from the house, and burned it. About nine days later the horse was taken sick with all the symptoms of scarlet fever. During her sickness the hair came off and the cuticle peeled as in the desquamation of scarlatina. Veterinary surgeons declare that horses do not have scarlet fever because they do not find the bacillus of that disease.

Dr. James—The horse of a patient of mine, who was a strong homœopath, as far as himself and wife went, was put in the hands of a veterinary. The horse was sick from a cold taken while driving, or rather from standing after driving. He called in a veterinary. He sent for a prominent veterinary physician, who said that as a horse was ten times heavier than a man he required ten times as much medicine. He accordingly gave him two hundred and fifty grains of Quinine, and at the third dose the horse fell dead in the stall.

Dr. Sawyer—Somebody asked whether there had been any cures of spavin. There have been several cures to my knowledge. A man whose wife I was treating for cancer of mammæ told me he thought Homœopathy was nothing but faith cure.

I noticed that his horse had some ugly fistulous openings, and asked him if he thought his horse had much faith, and told him I could cure his horse of those fistulous openings. I gave three powders of Silicea, and they got well. His wife also recovered.

Dr. J. H. Allen—We have a number of large horse farms in Indiana, where they raise blooded stock, the majority of the farmers treat them with homœopathic specifics and seldom lose a horse from sickness.

Dr. W. L. Morgan—About four years ago the stableman came after me, early in the morning, saying my horse was very sick. I found the horse's head stuck out straight and under the jaws and forelegs swollen so that he could not get his head to the ground. On inquiry I found that a horse in the same stable was having pinkeye and was under a veterinary. I gave Bell.$^{200}$ on the horse's tongue and put him in a box stall. In the evening I went to the stable again and I saw the horse was beginning to want to eat. We gave him a little green grass and he ate. In two days he was in condition to use.

Dr. Rushmore—A valuable cow in some way got loose in the stable, and thus had access to the ground feed barrel. When my attention was called to her she was lying prostrate on the ground. Eyes dull, tongue stretched out of her mouth and cold. I placed a few pellets of Carbo-veg.$^{200}$ on her tongue and she got well rapidly. I would not have risked a dollar on her chances of recovery.

Dr. Kent—I have just one dog story. This dog had become too much interested in a cow that was going through the process of labor. The dog persisted in his attention until the cow turned on him and hooked him through the hind leg. It was a punctured wound and stiffness followed its healing, so he was no longer able to pose as a ten-thousand-dollar dog. It seemed to be chronic stiffness and induration. I sent a dose of Ledum which restored him to usefulness and to his proper place as a prize dog.

